# Machine learning-driven discovery of celastrol as an anti-inflammatory therapy suppressing NETs in severe influenza

**DOI:** 10.1016/j.gendis.2025.101971

**Published:** 2025-12-09

**Authors:** Rui Gui, Lei Xue, Lele Li, Jianmei Xiao, Li Peng, Meng Ni, Wanling Peng, Haoliang Wang, Zhili Liu, Guohong Deng

**Affiliations:** a Department of Infectious Diseases, Southwest Hospital, Third Military Medical University (Army Medical University), Chongqing 400038, China; b Chongqing Key Laboratory of Viral Infectious Diseases, Chongqing 400038, China; c Yu-Yue Center for Pathology Research, Chongqing 400039, China; d Department of Obstetrics and Gynaecology, The Chinese University of Hong Kong, Hong Kong 999077, China; e Songjiang Research Institute, Shanghai Key Laboratory of Emotions and Affective Disorders, Songjiang Hospital Affiliated to Shanghai Jiao Tong University School of Medicine, Shanghai 200025, China

Glucocorticoids treat severe influenza but raise the risk of secondary infections, highlighting the need for new anti-inflammatory drugs. The inefficiency and cost of traditional drug discovery drive machine learning-guided discovery of targeted immunomodulators.^1^^,^^2^ We applied this strategy to screen candidate agents to improve survival in severe influenza.

To examine the influence of delaying antiviral administration on influenza treatment, C57BL/6 mice were treated with baloxavir at 0, 1, or 2 days post-inoculation (dpi) with influenza A virus (IAV) (Fig. S1A). Immediate treatment (0 dpi) ensured full survival (100% *vs*. 0% in DMSO), while a 2-day delay (2 dpi) greatly reduced efficacy (Fig. 1A). Furthermore, baloxavir administered at 2 dpi only partially recovered weight loss, clinical scores, and lung function (Fig. S1B–S1F). Additionally, cytology analysis of bronchoalveolar lavage fluid showed time-dependent inflammatory cell infiltration in delayed treatment groups, with elevated nucleated cell counts and red blood cell presence (Fig. S1G and S1H). Viral loads inversely correlated with treatment timing (Fig. S1I). Early antiviral initiation (0 dpi) significantly suppressed pro-inflammatory cytokine production in lung tissues compared with delayed treatment (Fig. S1J). Similarly, gross pathology revealed reduced hemorrhagic foci in the immediate treatment group (Fig. S1K). Finally, histological analysis revealed exacerbated hemorrhage and progressive fibrosis in infected lungs compared with the delayed treatment group (Fig. S1L–S1N).

**Figure 1:**
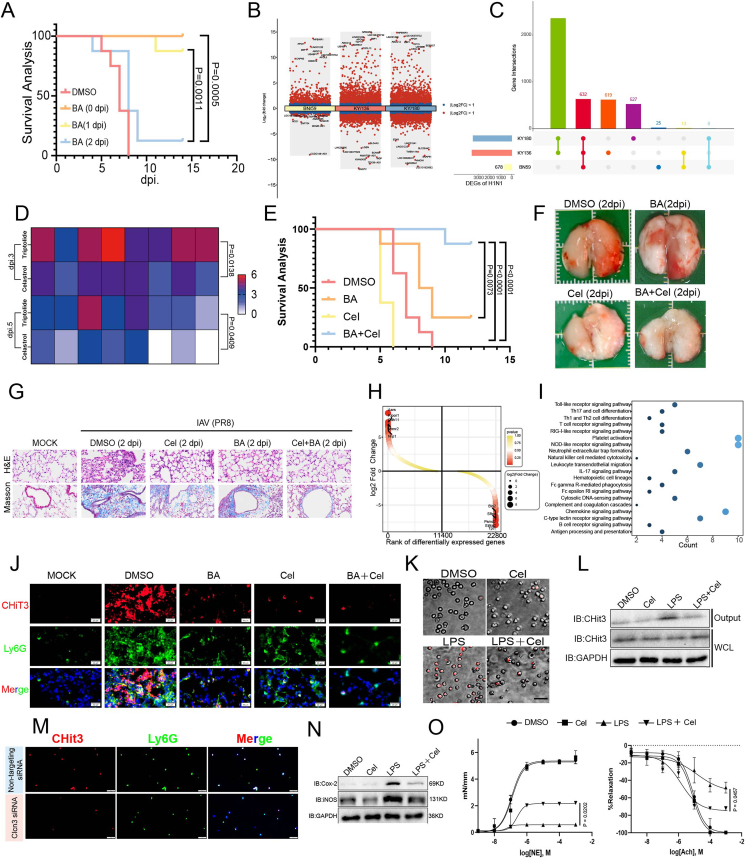
Celastrol-baloxavir synergy suppresses Clcn3-dependent NET formation to improve survival in severe influenza. **(A)** Survival kinetics of influenza A virus (IAV)-infected C57BL/6 mice treated with baloxavir at 0, 1, or 2 days post-inoculation (dpi) (*n* = 8). **(B)** The volcano plots showing differentially expressed genes (DEGs) for each viral strain (BN/59, KY/136, KY/180), highlighting significantly up-regulated and down-regulated genes. **(C)** The upset plot identifying 632 genes commonly affected by all three strains. **(D)** Clinical severity scores in mice treated with celastrol + baloxavir versus triptolide + baloxavir (*n* = 8). **(E)** Survival benefits of celastrol + baloxavir versus monotherapies (*n* = 8). **(F)** Gross observation of lungs at 5 dpi. **(G)** Histopathology of lungs at day 5 post-infection. Hematoxylin-eosin staining (G, upper) indicates increased injury, and Masson's staining (G, lower) visualizes fibrosis with indicated treatment. Scale bar, 50 μm. **(H)** Distinct transcriptional clustering between celastrol- and DMSO-treated mice by RNA sequencing (5 dpi). **(I)** Pathway analysis revealed significant enrichment in inflammatory pathways, including TLR, RIG-I-like receptor, NOD-like receptor, and NET formation in the vehicle group compared with celastrol treatment. **(J)** Representative images of lung infiltrating neutrophils (Ly6G, green) and presence of NETs (citrullinated H3, red) in mice infected with IAV for 5 days. Scale bar, 20 μm. **(K)** Neutrophils were pretreated with celastrol (3 μM, 2 h), stimulated with lipopolysaccharide (LPS, 20 μg/mL, 2 h), and imaged live using the IncuCyte system. Non-viable cells were stained with SYTOX Green (100 nM) and analyzed at the endpoint. Scale bar, 100 μm. **(L)** Western blot analysis of CHit3 in celastrol-treated (3 μM) NETs after being stimulated with LPS (20 μg/mL) for 3 h. **(M)** Immunofluorescence staining of Ly6G^+^ neutrophils (green) and citrullinated histone H3 (red) in CLCN3-knockdown versus control groups at 4 h post-LPS stimulation (20 μg/mL). Scale bar, 50 μm. **(N)** Western blot analysis of iNOS and COX-2 in endothelial cells after a 6-h co-incubation with NETs (20 μg/mL) derived from celastrol-treated (3 μM) neutrophils. Scale bar, 50 μm. **(O)** Celastrol (3 μM) restores *ex vivo* mesenteric artery contractility impaired by LPS-activated NETs. Statistical analysis was performed using the log-rank test for (A, E) and the Student's *t*-test for (D, O), and significant *p*-values are indicated on the graphs.

To identify anti-inflammatory drug targets against influenza, we performed transcriptomic analysis on primary human bronchial epithelial cells infected with three H1N1 variants (BN/59, KY/136, KY/180) versus uninfected controls. Comparative analysis identified 678 (BN/59), 3621 (KY/136), and 3524 (KY/180) differentially expressed genes (|log_2_fold-change|>1, adjusted *p* < 0.05), with 632 core differentially expressed genes shared across strains (Fig. 1B and C; Table S1). Hierarchical clustering demonstrated distinct separation between infected and control groups based on differentially expressed gene patterns (Fig. S2A–S2C). Functional analysis of shared differentially expressed genes revealed significant enrichment in inflammatory pathways (41 genes; *p* < 0.01), including acute/chronic inflammation and inflammasome assembly (Fig. S2D), supporting cytokine-mediated pathology as a therapeutic target. PROGENy pathway analysis identified Janus kinase/signal transducer and activator of transcription (JAK-STAT) signaling as the most significantly activated pathway across strains (Fig. S2E–S2G). Gene set enrichment analysis confirmed robust JAK-STAT activation (Fig. S2H–S2J). This analysis identifies conserved transcriptional responses, emphasizing epithelial inflammation and JAK-STAT signaling in host–virus interactions. Then, we applied weighted gene co-expression network analysis (WGCNA) with a soft-threshold *β* = 20 (scale-free *R*^2^ = 0.85), generating 14 co-expression modules (170–5916 genes/module; Table S2). Eigengene-trait correlation analysis linked seven modules (MEcyan, MEsalmon, MEbrown, MEred, MEgreen, MEtan, MEblue) to H1N1 infection (Fig. S3A). Hub genes (GS > 0.2, MM > 0.8) were identified for each infection-associated module (Fig. S3B–S3H), and their regulatory interplay was mapped (Fig. S3I). Functional annotation highlighted central roles of these modules in viral response and innate immune regulation. Finally, dual ridge regression models (CTRP/PRISM) predicted drug response using transcriptomes of H1N1-infected bronchial epithelial cells (strains: BN/59, KY/136, KY/180). Differential analysis of dose–response AUCs between infected and non-infected groups revealed strain-specific candidates: 18–25 candidates via CTRP and 20–33 via PRISM (Fig. S4A–S4H). Algorithmic cross-validation prioritized triptolide for anti-inflammatory potential. Upon CMAP integration, celastrol was the only compound effective across all three viral strains (Fig. S4I). *In vivo* celastrol-baloxavir outperformed triptolide-baloxavir in reducing IAV severity (Fig. 1D), prompting further mechanistic investigation of celastrol.

We next evaluated celastrol's effectiveness against severe influenza (Fig. S5A). The combination of celastrol and baloxavir improved survival (Fig. 1E) and accelerated weight recovery (Fig. S5B and S5C), accompanied by milder symptoms (Fig. S5D) and enhanced pulmonary function (Fig. S5E and S5F). Compared with monotherapies, the combination reduced lung inflammation (Fig. S5G and S5H) and damage (Fig. 1F), attenuating fibrosis and tissue injury (Fig. 1G; Fig. S5I and S5J). Furthermore, it suppressed pro-inflammatory cytokines without increasing viral titers (Fig. S5K and S5L).

To investigate the potential targets of celastrol, we conducted a separate analysis comparing the gene expression profiles of the celastrol-treated group to the vehicle (DMSO) group. Celastrol treatment induced two distinct gene expression patterns compared with vehicle (Fig. 1H). RNA sequencing of IAV-infected mouse lungs (5 dpi) demonstrated celastrol's vascular protective effects (Fig. S6A). Pathway analysis revealed that baloxavir alone suppressed fibrosis-related genes (Fig. S6B), whereas its combination with celastrol synergistically attenuated inflammation (Fig. S6C). Notably, celastrol alone exhibited insufficient protective anti-inflammatory effects, underscoring viral-induced damage as a critical early driver and the necessity of timely antiviral intervention. Gene expression profiling of celastrol-treated versus vehicle-treated mice revealed suppression of inflammatory pathways. Key mediators of IAV immunopathology, including Toll-like receptor (TLR), retinoic acid inducible gene-I (RIG-I), NOD-like receptor, and neutrophil extracellular trap (NET) formation pathways, were enriched in controls but suppressed by celastrol (Fig. 1I; Fig. S6D). Since NET formation is linked to lethal influenza outcomes, we focused on neutrophils.^2^ Single-cell RNA sequencing (GSM6304387) identified neutrophils as a major population (Fig. S6E), confirmed by CellMarker/PanglaoDB markers (Fig. S6F). Computational knockout (scTenifoldKnk) of celastrol-down-regulated genes (*Actg1*, *Clcn3*) disrupted neutrophil apoptosis and homeostasis networks (Fig. S6G and S6H), suggesting a mechanism underlying celastrol's NETosis inhibition.^3^

Consistent with our hypothesis that celastrol targets neutrophils, it significantly reduced NET formation *in vivo*, as evidenced by decreased chitinase 3-positive (CHit3^+^) signals adjacent to lymphocyte antigen 6 family member G-positive (Ly6G^+^) neutrophils (Fig. 1J; Fig. S7A and S7B) and reduced NETs in bronchoalveolar lavage fluid or plasma (Fig. S7C and S7D). In lipopolysaccharide-stimulated primary neutrophils (approximately 90% purity; Fig. S7E–S7G), celastrol significantly inhibited cell death (quantified by SYTOX® Green staining) (Fig. 1K; Fig. S7H). Additionally, it suppressed CHit3 expression in immunoblot analysis (Fig. 1L; Fig. S7I) and diminished Ly6G-CHit3 co-localization by immunofluorescence (Fig. S7J and S7K). Mechanistically, celastrol down-regulated chloride voltage-gated channel 3 (Clcn3) more strongly than actin gamma 1 (Actg1) in HL60-derived neutrophil-like cells (Fig. S7L and S7M), suggesting that CLCN3 dysregulation may be a key driver of NET suppression. Consistent with this, CLCN3 knockdown in HL60-derived neutrophil-like cells attenuated NETs (Fig. 1M; Fig. S7N, S7O), linking CLCN3 modulation to celastrol's anti-NET effect.

Since celastrol attenuated IAV-triggered NET formation and pulmonary hemorrhage, we next investigated whether this effect was mechanistically linked to vascular endothelial protection. Flow cytometry showed that celastrol reduced intercellular adhesion molecule 1 (ICAM-1) and E-selectin on endothelial cells (Fig. S8A–S8C). Meanwhile, immunoblotting revealed that NETs from celastrol-treated neutrophils suppressed inducible nitric oxide synthase (iNOS) and cyclooxygenase-2 (COX-2) expression (Fig. 1N; Fig. S8D). Microscopy further showed that NETs derived from celastrol-treated neutrophils maintained endothelial cell integrity (elongated, adherent morphology), whereas lipopolysaccharide-induced NETs triggered apoptotic features (cell shrinkage/rounding) (Fig. S8E). Crucially, *ex vivo* mesenteric artery assays confirmed that lipopolysaccharide-activated NETs severely impaired both contractility and endothelium-dependent relaxation, while celastrol treatment restored vascular function close to untreated control levels (Fig. 1O). These findings collectively demonstrate that celastrol-mediated vascular protection is mechanistically linked to NET inhibition.

Celastrol shows promise as an immunomodulator for severe influenza by suppressing Clcn3-dependent NETosis and limiting vascular injury. Clcn3 is critical for neutrophil functions, including phagocytosis, migration, and respiratory burst.^4^ Consistently, celastrol reduced pulmonary neutrophil recruitment and activated neutrophils (Fig. 1J), suggesting that Clcn3 may modulate NET generation via respiratory burst regulation.

Notably, celastrol was identified as a candidate anti-inflammatory drug for severe influenza through ridge regression-based computational screening, with subsequent transcriptomic analysis implicating Clcn3 as a putative target. Future studies could use AlphaFold-predicted models for *in silico* docking and optimization, refining celastrol's efficacy while minimizing off-target effects.^5^ This AI-integrated strategy combining screening, validation, and structure-based optimization may offer a pipeline for severe influenza and other hyperinflammatory syndromes.^5^

## CRediT authorship contribution statement

**Rui Gui:** Writing – review & editing, Writing – original draft, Visualization, Validation, Project administration, Methodology, Investigation, Formal analysis, Data curation, Conceptualization. **Lei Xue:** Validation, Methodology, Investigation. **Lele Li:** Validation, Methodology, Investigation. **Jianmei Xiao:** Methodology, Investigation. **Li Peng:** Methodology, Investigation. **Meng Ni:** Methodology, Investigation. **Wanling Peng:** Supervision, Project administration. **Haoliang Wang:** Supervision, Project administration, Conceptualization. **Zhili Liu:** Writing – review & editing, Writing – original draft, Visualization, Validation, Supervision, Software, Project administration, Methodology, Investigation, Formal analysis, Data curation, Conceptualization. **Guohong Deng:** Supervision, Project administration, Funding acquisition.

## Ethics declaration

All animal experiments were approved by the Animal Ethics Committee of Army Medical University (No. AMUWEC20242046) and conducted in an ABSL-2 laboratory.

## Funding

This work was supported by the Chongqing Natural Science Foundation Program (China) (No. CSTB2025NSCQ-GPX0651), Chongqing Medical Excellence Team (China), Yu-Yue Pathology Scientific Research Center (China) (No. YYKYXM202303AZ-202), and the 10.13039/501100001809National Natural Science Foundation of China (No. 82500021 and 81930061).

## Conflict of interests

The authors declared no competing interests.
