# Supplementary material for: Machine learning-driven discovery of celastrol as an anti-inflammatory therapy suppressing NETs in severe influenza
Source: Genes Dis. 2025 Dec 9;13(4):101971. doi: 10.1016/j.gendis.2025.101971 (PMC13015225; doi:10.1016/j.gendis.2025.101971)
Supplement: Multimedia component 1 [file mmc1.docx]

**Materials and methods**

**Identification of Differentially Expressed Genes**

We collected the RNA expression data of primary human bronchial epithelial cells infected with three different variants of H1N1 virus and their control samples from Gene Expression Omnibus (GEO) database (https://www.ncbi.nlm.nih.gov/geo/) (Series: GSE48466, Samples: GSM1179364, GSM1179365, GSM1179366, GSM1179367, GSM1179368, GSM1179369, GSM1179370, GSM1179371, GSM1179372, GSM1179373, GSM1179374, GSM1179375). BN/59, KY/136 and KY/180 are three variants of H1N1 virus in the data set. The primary human bronchial epithelial cells were infected by BN/59, KY/136 and KY/180 for 36 hours in vitro. Each strain and control group infection were performed with three biological replicates. And then, the extraction of total RNA was performed. The limma R package (version 3.52.2) was utilized for performing differential expression analysis, and the criteria for differentially expressed genes (DEGs) were set as |log_2_ fold change| > 1 and adjusted *p*-value < 0.05.

**Functional Enrichment Analysis**

The biological process of Gene Ontology (GO) analysis and Gene Set Enrichment Analysis (GSEA) were conducted using R package clusterProfiler (version 4.8.1). R package PROGENy (version 1.22.0) and decoupleR (version 2.6.0) were used to estimate pathway activity changes.

**Weighted Gene Co-Expression Network Analysis**

Weighted gene co-expression network analysis (WGCNA), a prevalent algorithm in systems biology, is frequently employed to investigate the intricate relationships between gene sets and phenotypic traits by building free-scale gene co-expression networks/modules. In this study, we utilized the mRNA expression profile to construct a gene co-expression network using the WGCNA package implemented in R software, version 1.72. The critical WGCNA::blockwiseModules() function operated under a refined set of parameters, including a soft threshold power of 20, TOMType specified as "unsigned", a minimum module size criterion of 100, deepSplit value set to 3, and mergeCutHeight at 0.25.

**Predicting Potential Therapeutic Agents**

The CTRP and PRISM datasets encompass gene expression profiles and drug sensitivity data for numerous human cancer cell lines, with drug sensitivity evaluated using area under the dose-response curve (AUC) values. Lower AUC values indicate greater sensitivity to a given treatment. Utilizing these datasets, we developed a ridge regression model through the pRRophetic package (version 0.5) to analyze drug response patterns in H1N1-infected samples. This approach allowed us to estimate AUC values for individual compounds across samples, offering insights into their potential therapeutic efficacy.

In addition, we employed the cluequery R package (a strategy based on perturbation) to predict the candidate agents. This method identifies drugs or drug combinations predicted to reverse or mimic specific gene expression signatures. By combining these two approaches, we systematically screened potential therapeutic agents for H1N1 infection.

**Virtual gene knockout**

The scTenifoldKnk R package was used to perform virtual gene knockout experiment in silico. It is an efficient virtual gene knockout tool based on machine learning workflow. We could infer the effects of gene knockout in single-cell data via the software.

**RNA-Seq and Analysis**

RNA sequencing was conducted by Wuhan IGENEBOOK Biotechnology Co., Ltd(http://www.igenebook.com). Total RNA was extracted using the Trizol method, and RNA integrity was evaluated with a Qsep400 instrument. RNA libraries were prepared using the VAHTS mRNA-seq V8 Library Prep Kit for Illumina, starting with 1 μg of total RNA. The workflow included poly(A) RNA enrichment, fragmentation, reverse transcription with random hexamers, and 150 bp paired-end sequencing on an Illumina NovaSeq 6000 platform.

**Animal experiments**

Mice were anesthetized via intraperitoneal injection of Avertin and subsequently infected intranasally with 100 plaque-forming units (PFU) of the virus in a total volume of 40 μl. Control mice were administered an equivalent volume of physiological saline. The viral titer (PFU) was determined by plaque assay using MDCK cells after serial dilutions of the virus stock. Following infection, mice were monitored daily for weight loss and survival for up to 14 days or until clinical end point. Baloxavir marboxil (1985606-14-1, MCE) was administered daily via oral gavage at a dose of 10 mg/kg, while celastrol (34157-83-0, MCE) was administered every other day via intraperitoneal injection at a dose of 1 mg/kg. Control mice received an equivalent volume of PBS. Additionally, a clinical scoring system was employed to evaluate the health status of the mice over the course of the experiment. Mice were considered to have reached the clinical endpoint upon losing 30% of their initial body weight. The clinical scoring was based on the following criteria: (1 point each for) piloerection, hunched posture, partially closed eyes, labored breathing, reduced activity, and movement only when provoked; or (2 points for more severe manifestations).

**Pulmonary function tests**

Pulmonary function was evaluated using a whole-body plethysmography system (Data Sciences International), following established protocols. In brief, mice were individually placed in unrestrained plethysmography chambers, and after a 10-minute acclimatization period, respiratory parameters were recorded over a 30 minute duration using FinePointe software (Data Sciences International).

**Analysis of BALF**

The lungs were lavaged with two consecutive washes of 1 ml PBS each. The bronchoalveolar lavage fluid (BALF) was centrifuged at 300 g for 5 minutes at 4°C, and the supernatant was carefully collected and stored at -80°C for subsequent analysis. The cell pellet was resuspended in 1 ml PBS, and total leukocyte counts and red blood were determined by counting at least 100 leukocytes after staining with Giemsa, following the manufacturer’s instructions.

**Viral titer determination**

The fully confluent monolayer of MDCK cells was washed twice with PBS and then inoculated with the viral supernatant. After incubating for 1 hour, the cells were washed twice with PBS again. Subsequently, 1 × plaque maintenance solution (DMEM containing 1% FBS, 1% low melting point agarose, and 2 μg/mL TPCK-treated trypsin) was added. Once the plaque maintenance solution solidified completely, the plates were inverted and incubated at 37°C for 72 hours. Finally, the plaques were counted after staining with 1% crystal violet.

**Cytokine and chemokine analysis**

The level of Cytokines in BALF and plasma were detected by using Luminex Multi-factor Detection Technology (Abclonal, MCYTOMAG-70K) according to the manufacturer’s instructions. For heatmap visualization of expression profiles, normalized values were processed using GraphPad Prism version 9.0.

**Flow Cytometry**

Mouse aortic endothelial cells (MAECs) obtained from Wuhan Procell were utilized to investigate endothelial dysfunction markers, including vascular cell adhesion molecule-1 (VCAM-1, A23398, Abclonal), intercellular adhesion molecule-1 (ICAM-1, A22312, Abclonal), and E-selectin (A26630, Abclonal). These markers were assessed via flow cytometry following stimulation with 10 µg/ml NETs for 6 hours. The endothelial cells were digested using 0.25% trypsin and centrifuged at 300 × g for 5 minutes. Subsequently, the cells were incubated with 7-AAD (Invitrogen, 00-6993-50) and various fluorochrome-conjugated antibodies for 30 minutes at 4°C under dark conditions. After incubation, the cells were washed twice with PBS, resuspended in 200 µl PBS, and filtered through a 200-mesh nylon filter. Flow cytometric analysis was performed using a BD FACSCanto II instrument (BD Bioscience).

**Histology and** **Immunofluorescence (IF) Assay**

Excised lung tissues were fixed in 4% paraformaldehyde, sectioned, and stained with hematoxylin-eosin (H&E) and Masson's trichrome for histological analysis. Gross and microscopic pathological assessments were performed, and histopathological scoring was conducted by two professional veterinary pathologists based on the Smith system. Scores were assigned as follows: 0 (no lesions), 1 (lesion area ＜25%), 2 (lesion area 25-50%), 3 (lesion area 50-75%), and 4 (lesion area ＞75%). Lung fibrosis was quantified using the particle analyzer tool in FIJI ImageJ software.

Lungs from different groups of mice were collected and fixed in 4% paraformaldehyde for 24 hours, followed by dehydration in 30% sucrose for an additional 24 hours. The entire lungs were then frozen and sectioned into 8 μm thick slices using a cryostat. The sections were permeabilized at room temperature with 0.1% Triton X-100 and subsequently blocked with goat serum for 2 hours at room temperature. The primary antibodies, Anti-CitH3 (1:400, ab5103, Abcam) and Anti-Ly6G (1:400, ab25377, Abcam), were added and incubated overnight at 4°C. After incubation with the primary antibodies, the sections were treated with FITC-conjugated Goat Anti-Rat (1:400, SA00003-11, Proteintech) and Cy3-conjugated Goat Anti-Rabbit (1:400, A0516, Beyotime) for 2 hours at room temperature. Finally, the sections were counterstained with 4′,6-diamidino-2-phenylindole (P36931, Thermo Fisher Scientific) and examined using an Olympus VS200 scanner. The total areas of Ly6G and CitH3 were quantified using the particle analyzer tool in Fiji image analysis software

Before use, the cell culture slides were sterilized with ultraviolet light and positioned in a 12-well plate. Purified neutrophils (2 × 10^5 cells) were seeded into each well and allowed to adhere for approximately 10 minutes before initiating experimental treatments. Following treatment, the cells were fixed at room temperature with 4% paraformaldehyde for 2 hours and stained using the same protocol as for frozen sections. After staining, a custom hook, fashioned by bending the tip of an insulin syringe, was used to carefully retrieve the slides. The slides were then mounted in a medium containing 4′,6-diamidino-2-phenylindole (DAPI, P36931, Thermo Fisher Scientific) and observed with an Olympus VS200 scanner.

**Isolation and purification of neutrophils**

Femurs and tibias were harvested from 6-week-old C57BL/6J mice, and bone marrow was subsequently isolated. The cells were then resuspended in sterile Ca²⁺/Mg²⁺-free PBS. Neutrophils were isolated using negative selection with magnetic beads according to the manufacturer's instructions. The viability and purity of the isolated neutrophils were verified using flow cytometry. Specifically, cell viability was assessed using 7-VADD staining, while the markers CD11b (A24095, Abclonal) and Ly6G (PE-65140, Proteintech) were employed to characterize the purity of the neutrophil population.

**Western Blot Analysis**

Cells were lysed in RIPA buffer after being washed with cold PBS. The proteins were then separated using 10% polyacrylamide gels and transferred onto polyvinylidene difluoride (PVDF) membranes. The membranes were blocked with 5% skim milk and subsequently incubated with specific primary antibodies, followed by horseradish peroxidase (HRP)-conjugated secondary antibodies: Anti-CitH3 (1:1500, ab5103, Abcam); Anti-iNOS (1:1500, Abclonal, A25899); Anti-COX2 (1:1500, Abclonal, A3560).

**Real-time cell death analysis**

Cell death was monitored in real-time using the Incucyte Live-Cell Analysis System, following the manufacturer's protocol. Briefly, neutrophils were seeded in 96-well plates (2×10⁵ cells/well) and treated with celastrol (3 μM). After 2 hours, SYTOX Green (100 nM, Thermo Fisher Scientific, S7020) was added to label dead cells, followed by stimulation with LPS (20 μg/ml). Images were acquired and analyzed using IncuCyte S3 software.

**Quantitative real-time PCR**

The mRNA levels were quantified using real-time reverse transcription PCR (RT–qPCR) on a QuantStudio 3 Real-Time PCR System (Thermo Scientific) with the SYBR Green fluorescence detection kit (R222-01, Vazyme). Total RNA was extracted from treated MAECs using the RNA extraction kit (R4130, mgenbio) following the manufacturer's protocol. For cDNA synthesis, 1 μg of total RNA was reverse-transcribed using the HiScript III Reverse Transcriptase Kit (R333, Vazyme). The primers used for mouse *Gapdh*, *Actg1*, and *Clcn3* were as follows:

*Gapdh*: Forward: 5′-AGGTCGGTGTGAACGGATTTG-3′, Reverse: 5′-GGGGTCGTTGATGGCAACA-3′; *Actg1*: Forward: 5′-AATCGCCGCACTCGTCATT-3′, Reverse: 5′-GCCCTACGATGGAAGGGAA-3′; *Clcn3*: Forward: 5′-GACTGGGTGCGAGAGAAGTG-3′, Reverse: 5′-CATCCTGACCAGGCGTCATAC-3′. Each reaction was performed in triplicate, and relative mRNA levels were calculated using the 2^^-ΔΔCt^ method, with *Gapdh* as the internal control.

**RNA Interference**

The small interfering RNA (siRNA) targeting *CLCN3* and the negative control (NC) siRNA were synthesized by Sangon Biotech (Shanghai, China) using the same sequences as reported previously. The sense and antisense strands for *CLCN3* siRNA were 5′-CAAUGGAUUUCCUGUCAUATT-3′ and 5′-UAUGACAGGAAAUCCAUUGTA-3′, respectively. Successful transfection and gene silencing in HL60 cells were confirmed via western blot analysis.

**NETs collection**

A modified protocol was employed to isolate NETs released by LPS-stimulated neutrophils. Briefly, bone marrow-derived neutrophils were purified and seeded into 6-well culture plates at a density of 3 × 10⁶ cells/ml in RPMI 1640 medium supplemented with 2% FBS. Cells were incubated under standard culture conditions (37°C, 5% CO₂) and stimulated with LPS (20 μg/ml) for 4 hours.

Following stimulation, the culture supernatants were carefully aspirated, and the wells were gently washed twice with pre-cooled calcium- and magnesium-free PBS on ice to collect NETs-containing wash solutions. The collected solutions were centrifuged at 4°C, 300 × g for 5 minutes to remove cellular debris. The supernatants were then subjected to ultracentrifugation at 4°C, 25,000 × g for 20 minutes to pellet the NETs. The resulting pellet was resuspended in RPMI 1640 containing 1% FBS, and NETs concentration was quantified by measuring the absorbance at OD260 using a NanoDrop 2000 spectrophotometer.

**Vascular reactivity**

Twelve-week-old healthy C57BL/6J mice were sacrificed via Avertin administration (10 μl/g, i.p.) and immediately immersed in pre-cooled Krebs-Henseleit solution (130 mM NaCl, 3.7 mM KCl, 1.2 mM MgSO4, 1.2 mM KH2PO4, 25 mM NaHCO3, 2.5 mM CaCl2, 10 mM glucose). Under a dissecting microscope, the mesenteric arteries were carefully isolated and placed in a 6-well plate for stimulation with NETs (30 mg/ml) for 3 hours. After stimulation, arterial rings approximately 1.5 mm in length were transferred to organ baths and mounted on tungsten wires (0.04 mm diameter).

The arterial rings were equilibrated for 30 minutes in a water bath aerated with 95% O2 and 5% CO2. Standardization involved two successive contractions induced by a combination of KCl depolarization and noradrenaline (Y0000682, Sigma) to measure the vessels' maximum contractile capacity. After washing with Krebs-Henseleit solution and re-equilibration, cumulative concentrations of noradrenaline were applied (from low to high) to construct concentration-response curves.

Following another washing step and equilibration, the vessels were pre-contracted with noradrenaline (0.3 μM) to 90% of the maximum contraction. Cumulative concentrations of acetylcholine (2260-50-6, Sigma) were then added to generate concentration-response curves for acetylcholine-induced relaxation.

**Statistical analysis**

Data are presented as mean ± s.e.m. Statistical analyses were performed using GraphPad Prism 9, with the specific tests for each experiment indicated in the corresponding figure legends. A *P*-value of <0.05 was considered statistically significant.
