# Supplementary material for: Machine learning-driven discovery of celastrol as an anti-inflammatory therapy suppressing NETs in severe influenza
Source: Genes Dis. 2025 Dec 9;13(4):101971. doi: 10.1016/j.gendis.2025.101971 (PMC13015225; doi:10.1016/j.gendis.2025.101971)
Supplement: Multimedia component 2 [file mmc2.docx]

**Supplementary Figure Legends**


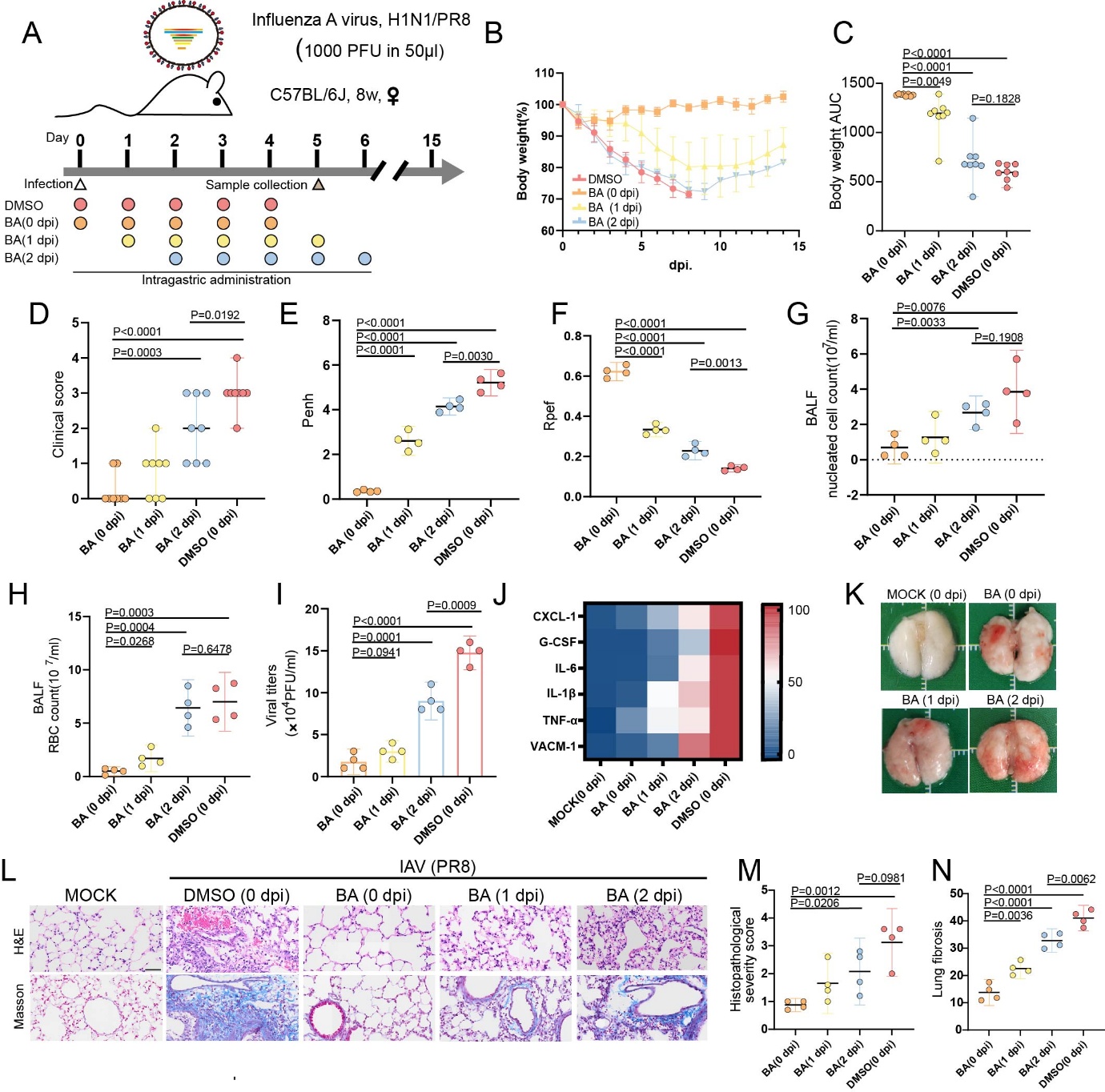


**Figure S1**: **Effectiveness of immediate and delayed treatment with the antiviral drug Baloxavir in a severe influenza virus infection mouse model.** (A) Experimental design: C57BL/6 mice inoculated with influenza A virus (IAV) received baloxavir or vehicle (DMSO) at 0, 1, or 2 days post-inoculation (dpi). (B, C, D) Body weight changes, area under the curve (AUC) of body weight over time in (B), and clinical scores of 6- to 8-week-old WT mice intranasally infected with 1000 PFUs of IAV and treated with DMSO or baloxavir. (E, F) Enhanced pause (Penh) and ratio of peak expiratory flow (Rpef) in mice at 5 dpi, measured using whole-body plethysmography. (G, H) Quantification of nucleated and red blood cells in BALF samples from different treatment groups at 5 dpi. (I) Viral titers in the BALF of infected mice. (J) Profile of G-CSF, IL-1β, TNF-α, VCAM-1, IL-6, and CXCL-1 in BALF from infected mice in different treatment groups at 5 dpi. (K) Gross examination of lungs at 5 dpi. (L-N) Histopathology of lungs: H&E staining (L, upper) indicates increased injury and Masson’s staining (L, lower) visualizes fibrosis (blue) with delayed baloxavir treatment. Quantitative histopathology scores (M) and fibrosis(N). Statistical analysis was performed using student’s t test (C-I, M-N), and significant *P* values are indicated on the graphs.


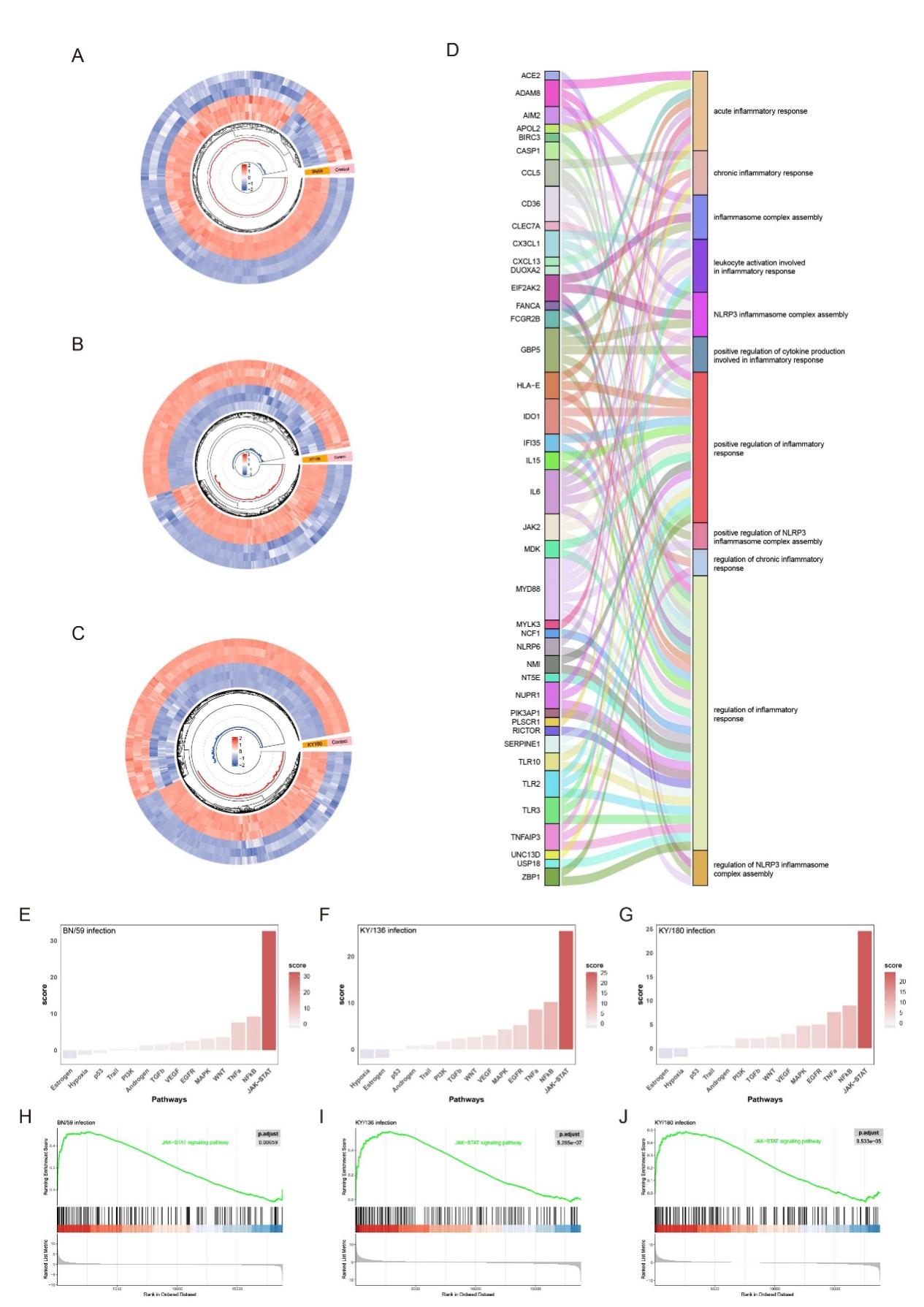


**Figure S2: Comprehensive Transcriptomic and Pathway Analysis of Influenza A Virus-Infected Primary Bronchial Epithelial Cells.** **(A-C)** Hierarchically clustered heatmaps of differentially expressed genes (DEGs, |log2FC|>1, adjusted. p<0.05) in bronchial epithelial cells infected with H1N1 variants. B: BN/59 (n=3) vs. controls (n=3) C: KY/136 (n=3) vs. controls D: KY/180 (n=3) vs. controls. **(D)** Functional enrichment analysis of shared DEGs, highlighting key inflammation-related pathways, including acute and chronic inflammatory responses and inflammasome assembly. **(E, F, G)** PROGENy pathway analysis indicating activation of the JAK-STAT signaling pathway in response to viral infection. **(H, I, J)** KEGG gene set enrichment analysis (GSEA) identifying key signaling pathways, providing insights into the molecular mechanisms underlying infection-driven transcriptomic changes.

**
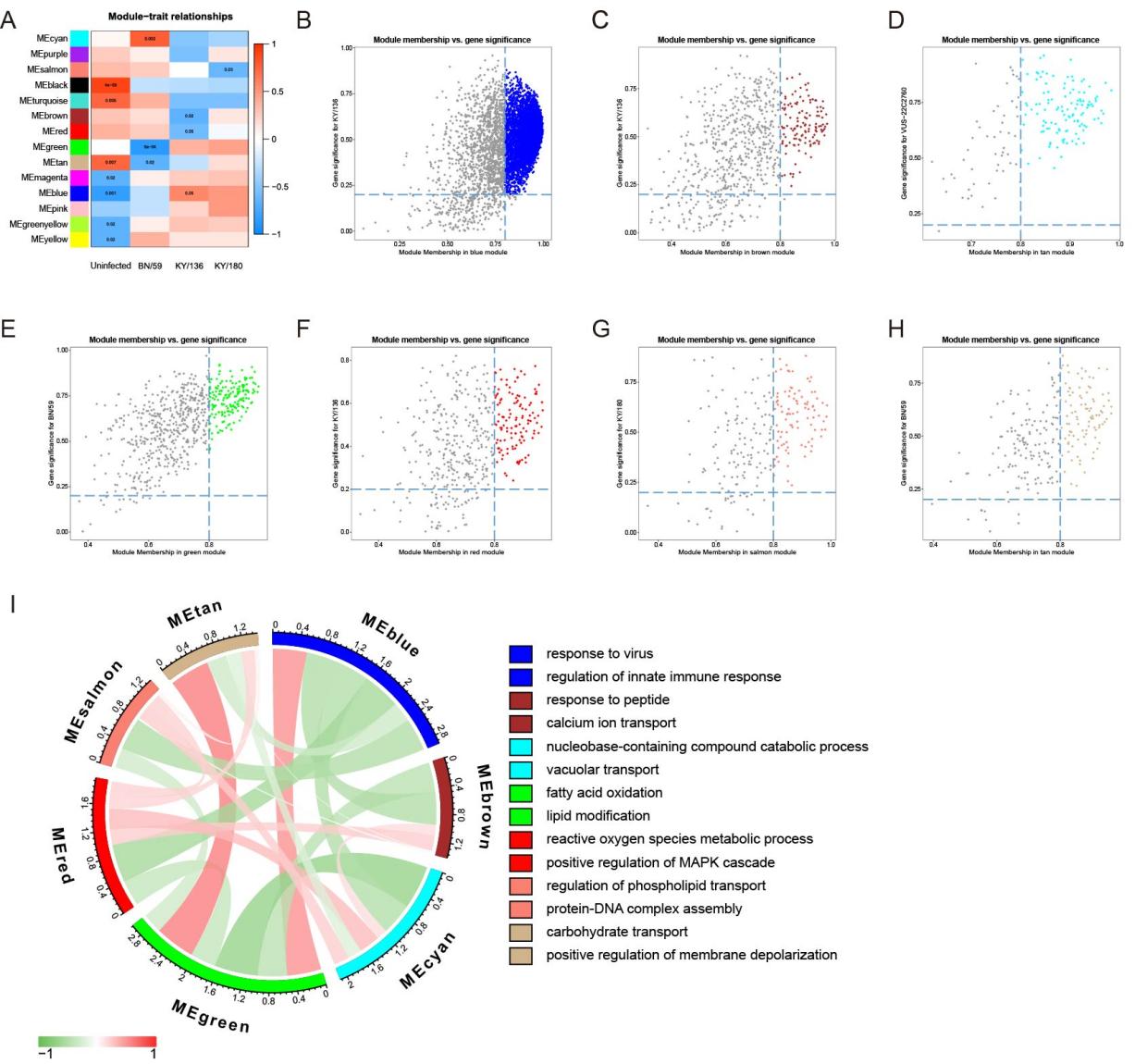
**

**Figure S3: Co-expression Modules Associated with H1N1 Infection and Identification of Potential Therapeutic Agents.** **(A)** Co-expression modules identified through Weighted Gene Co-expression Network Analysis (WGCNA) reveal strong correlations with H1N1 infection, highlighting seven key modules: MEcyan, MEsalmon, MEbrown, MEred, MEgreen, MEtan, and MEblue. **(B-H)** Hub genes for each module were selected based on gene significance (GS > 0.2) and module membership (MM > 0.8). **(I)** Correlation matrix of the modules with functional annotations, demonstrating their biological relevance to H1N1 infection.


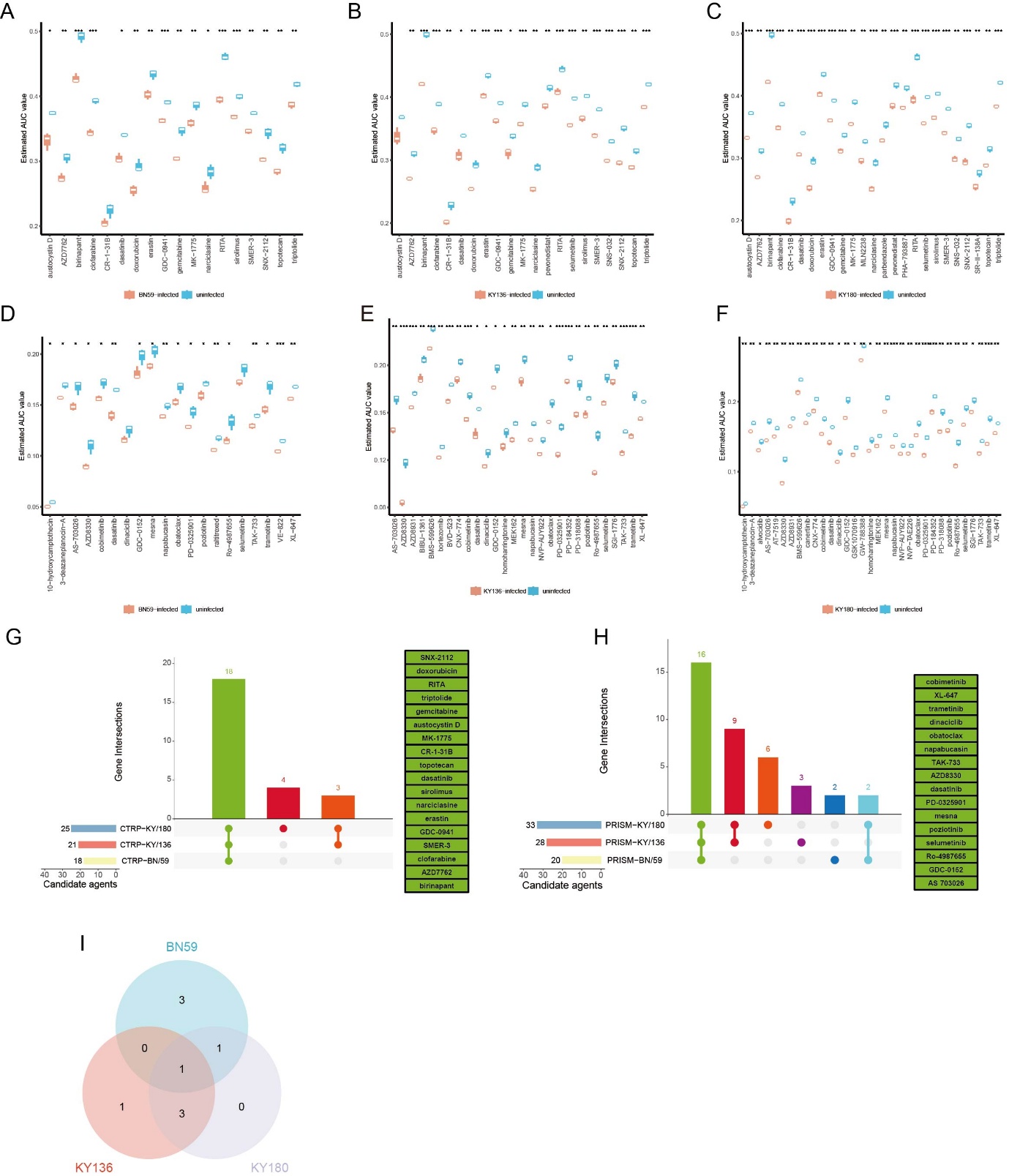


**Figure S4:** **Computational prediction of anti-influenza candidates through multi-database integration. (A-F)** Ridge regression-derived drug sensitivity profiles. CTRP-based prediction showing 18 (BN/59) (A), 21 (KY/136) (B), and 25 (KY/180) (C) candidates with significant AUC reduction. PRISM-derived prediction identifying 20 (BN/59) (D), 28 (KY/136) (E), and 33 (KY/180) (F) compounds. **(G-H)** Upset plots depicting strain-overlapping candidates. 18 consensus CTRP candidates (G), 16 shared PRISM candidates (H). **(I)** CMAP-integrated pan-strain drug prioritization. Strain-specific candidates were identified by correlating influenza-induced transcriptional profiles with CMAP-derived drug perturbation signatures.


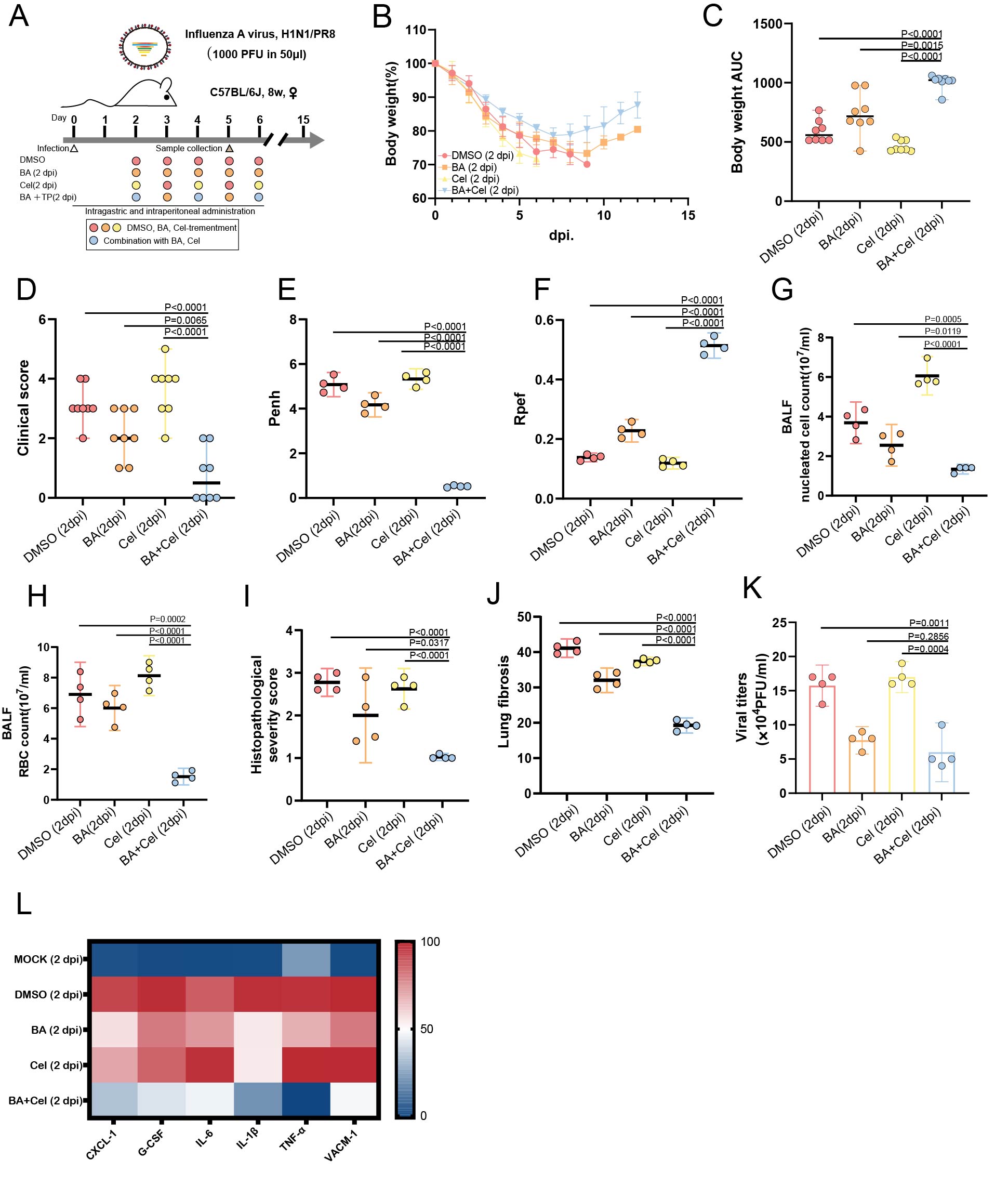


**Figure S5: Therapeutic validation of celastrol combined with baloxavir in influenza-infected murine models.** **(A)** Experimental design of diagram. (**B-C**) Accelerated weight recovery kinetics in celastrol + baloxavir group versus monotherapy/vehicle controls. **(D)** Clinically relevant symptom severity scoring following established metrics. **(E-F)** Pulmonary functional assessment through enhanced pause (Penh) and peak expiratory flow ratio (Rpef) measurement. **(G-H)** Bronchoalveolar lavage fluid (BALF) nucleated cell/RBC quantification showing inflammation mitigation. **(I-J)** Representative histopathology (H&E) and Masson’s staining quantification of lung injury and pulmonary fibrosis. **(K-L)** BALF viral loads and inflammatory cytokines by Luminex. Statistical analysis was performed using student’s t test (C-K), and significant *P* values are indicated on the graphs.


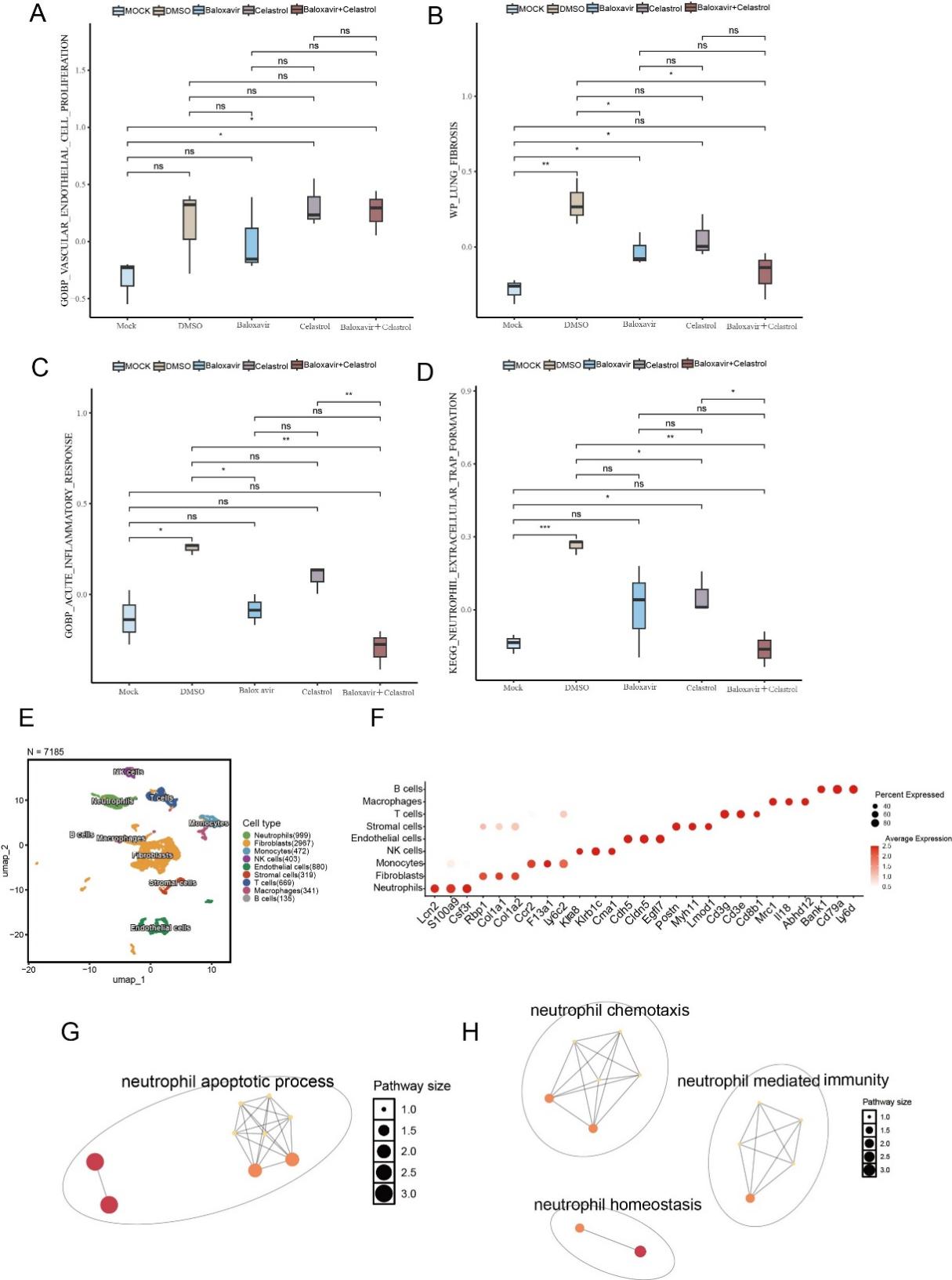


**Figure S6: Impact of celastrol and baloxavir on lung injury, inflammation, and fibrosis.** **(A)** Gene expression analysis of vascular injury markers showing protective effects of celastrol, but not baloxavir. **(B-D)** Pathway enrichment analysis revealing enhanced anti-fibrotic activity with baloxavir treatment (B), inflammatory cytokine levels in BALF showing reduced inflammation with combination therapy (celastrol + baloxavir) (C), activation of inflammatory pathways, including NETs formation reduced, following celastrol treatment (D). **(E)** Single-cell RNA sequencing identified nine major immune cell types from IAV-infected mice. **(F)** Validation of cell type annotation using marker genes confirmed accurate classification. **(G-H)** Virtual knockout of ***Actg1***(G) and ***Clcn3***(H) using scTenifoldKnk suggested impacts on neutrophil apoptosis and homeostasis, highlighting their potential role in celastrol's effects on neutrophils and NETs formation. The boxplot displays the P-values, with NS indicating no significance. The significance levels are as follows: **P* < 0.05, ***P* < 0.01, and ****P* < 0.001.


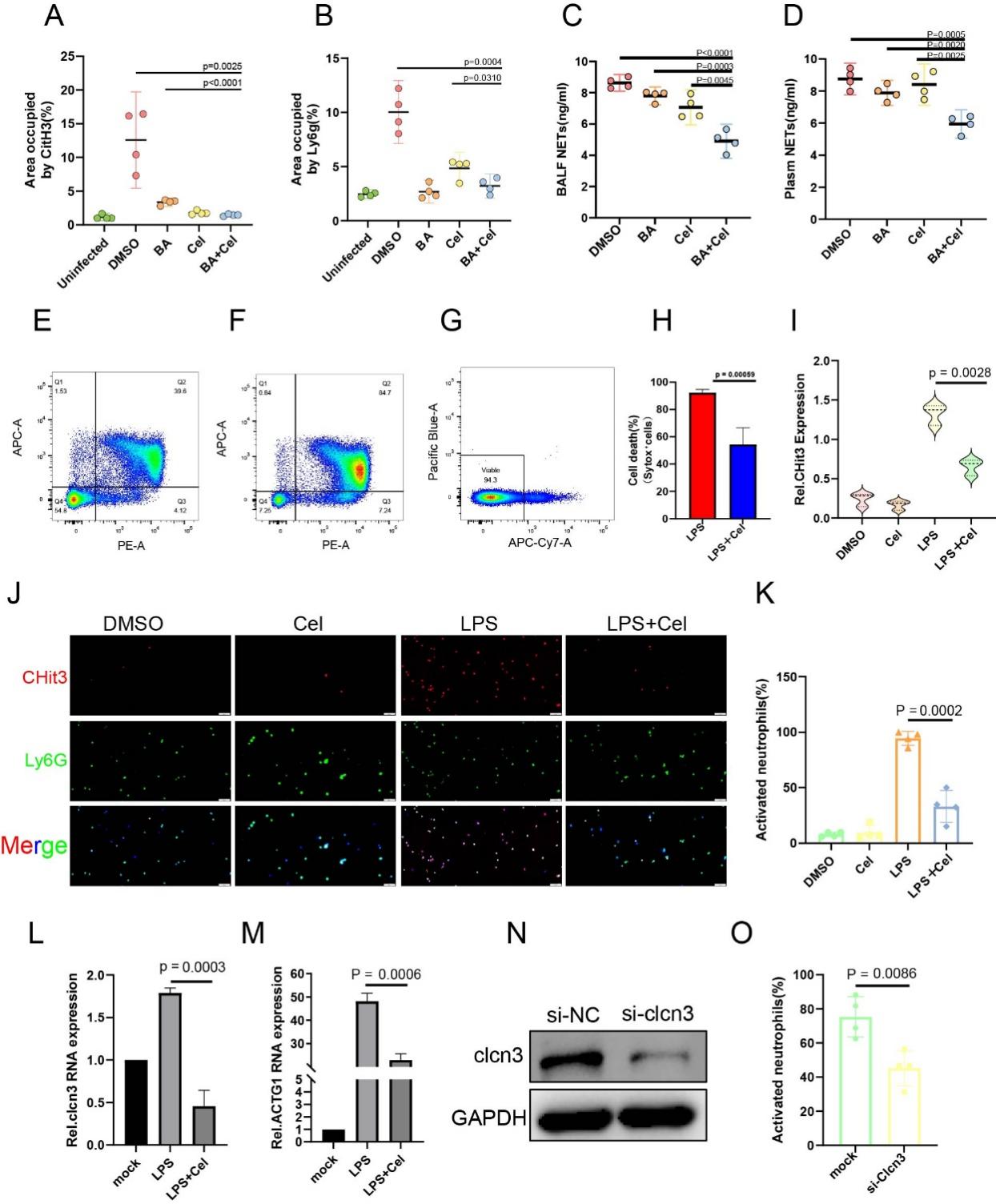


**Figure S7: Celastrol suppresses neutrophil extracellular traps (NETs) in vitro and in vivo. (A-B)** NET formation in lung tissues of IAV-infected mice treated with celastrol, quantified via Ly6G (green) and CHit3 (red) co-staining. **(C-D)** Reduced NETs levels in bronchoalveolar lavage fluid (BALF) and plasma by ELISA**. (E)** Representative flow cytometry plots of unpurified neutrophils stained with anti-CD11b and anti-Ly6G antibodies**. (F)** Neutrophils after purification via negative immunomagnetic selection, analyzed for purity using CD11Bband Ly6G staining. **(G)** Viability assessment of purified neutrophils using 7-AAD staining. **(H)** Quantification of the cell death observed in Figure1K. **(I)** Quantification of expression of CHit3 relative to GAPDH in the different treatment groups in Figure1L. **(J)** Immunofluorescence analysis of neutrophils (Ly6G, green) and NET formation (citrullinated H3, red) in the indicated groups at 2 hours post-stimulation. **(K)** Quantification of Ly6G⁺/CHIT3⁺ co-localized cells was observed in (J). **(L-M)** RT-qPCR analysis mRNA levels of Clcn3(H) and Actg1(I) in celastrol-treated neutrophils. **(N)** HL60 cells differentiated into neutrophil-like cells with 1.5% DMSO and transfected with siRNA to knock down *CLCN3*. Knockdown efficiency by siRNA transfection (48 hours), confirmed via immunoblotting. **(O)** Quantification of NETs calculated as co-localized CHit3^+^/Ly6G^+^ area percentage. Statistical analysis was performed using student’s t test (A-D, H-I, K, L-M, O), and significant *P* values are indicated on the graphs.


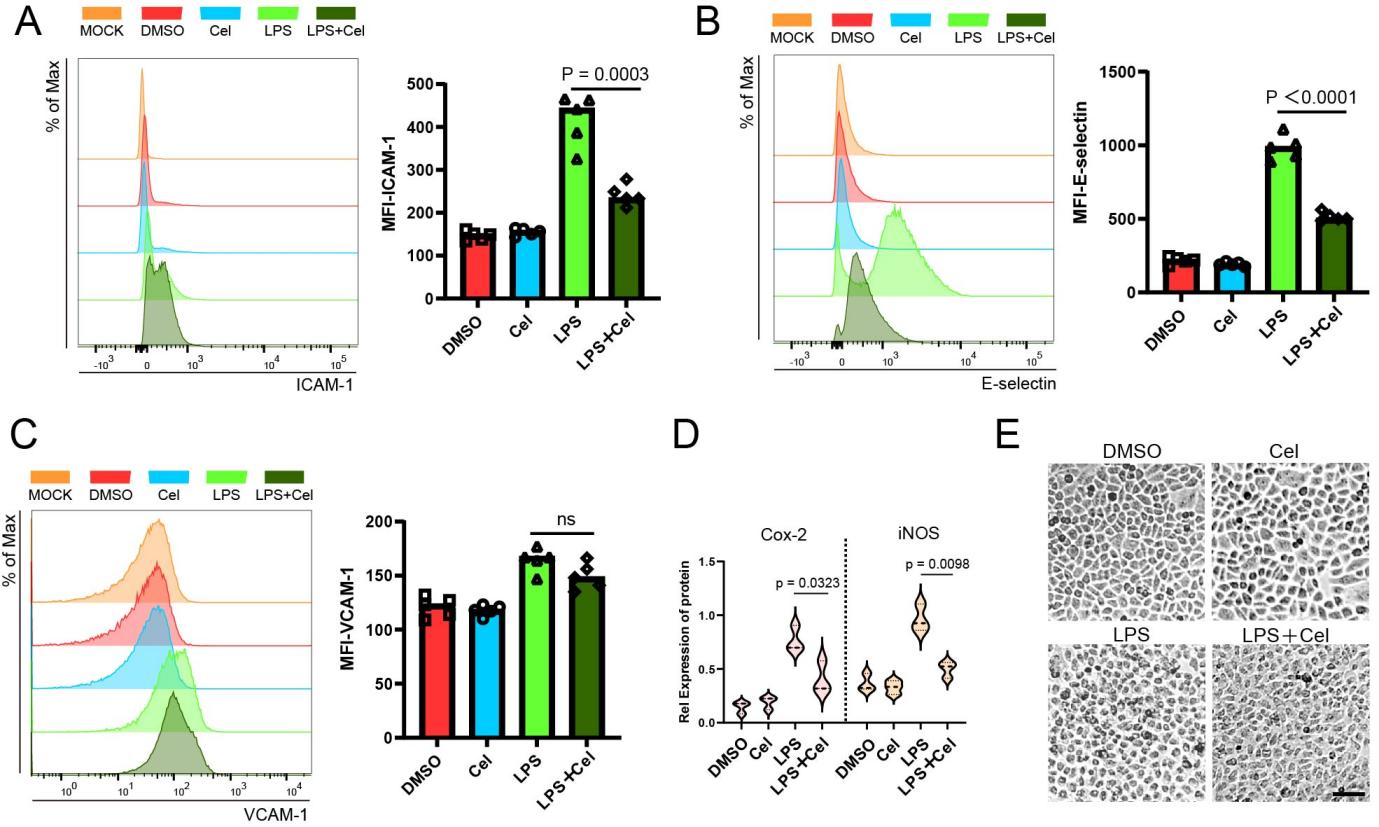


**Figure S8. Celastrol Protects Against NETs-Mediated Vascular Endothelial Injury.**

**(A-C)** Flow cytometric evaluation of endothelial cells co-cultured with NETs (neutrophil extracellular traps) generated from stimuli-treated neutrophils under indicated conditions. **(D)** Immunoblot analysis of iNOS and COX-2 expression in endothelial cells exposed to purified NETs, with quantification of protein levels normalized to GAPDH. **(E)** Microscopic assessment of endothelial cells treated with NETs. Scale bars, 10 μm. Statistical analysis was performed using student’s t test (A-D), and significant *P* values are indicated on the graphs.
